# Impact of end-of-life respiratory modalities on quality of dying and death and symptom relief in patients with interstitial lung disease: a multicenter descriptive cross-sectional study

**DOI:** 10.1186/s12931-022-02004-x

**Published:** 2022-04-04

**Authors:** Takafumi Koyauchi, Yuzo Suzuki, Kazuki Sato, Hironao Hozumi, Masato Karayama, Kazuki Furuhashi, Tomoyuki Fujisawa, Noriyuki Enomoto, Yutaro Nakamura, Naoki Inui, Koshi Yokomura, Shiro Imokawa, Hidenori Nakamura, Tatsuya Morita, Takafumi Suda

**Affiliations:** 1 Second Division, Department of Internal Medicine, Hamamatsu University School of Medicine, 1-20-1 Handayama, Higashi Ward, Hamamatsu, Shizuoka, 431-3192 Japan; 2 Nursing for Advanced Practice, Division of Integrated Health Sciences, Nagoya University Graduate School of Medicine, Aichi, Japan; 3 Department of Respiratory Medicine, Respiratory Disease Centre, Seirei Mikatahara Hospital, Shizuoka, Japan; 4 Department of Respiratory Medicine, Iwata City Hospital, Shizuoka, Japan; 5 Department of Respiratory Medicine, Seirei Hamamatsu Hospital, Shizuoka, Hamamatsu, Japan; 6 Department of Palliative and Supportive Care, Palliative Care Team and Seirei Hospice, Seirei Mikatahara Hospital, Shizuoka, Japan

**Keywords:** Quality of life, Pulmonary fibrosis, Interstitial Lung Disease

## Abstract

**Background:**

Respiratory modalities applied at the end of life may affect the burden of distressing symptoms and quality of dying and death (QODD) among patients with end-stage interstitial lung disease (ILD); however, there have been few studies into respiratory modalities applied to these patients near death. We hypothesized that high-flow nasal cannula (HFNC) might contribute to improved QODD and symptom relief in patients with end-stage ILD.

**Objectives:**

This multicenter study examined the proportion of end-of-life respiratory modalities in a hospital setting and explored its impact on QODD and symptom relief among patients dying with ILD.

**Methods:**

Consecutive patients with ILD who died in four participating hospitals in Japan from 2015 to 2019 were identified and divided into four groups according to end-of-life respiratory modality: conventional oxygen therapy (COT), HFNC, non-invasive ventilation (NIV), and invasive mechanical ventilation (IMV). In addition, a mail survey was performed to quantify the QODD and symptom relief at their end of life from a bereaved family’s perspective. QODD and symptom relief were quantified using the Good Death Inventory (GDI) for patients with a completed bereavement survey. The impact of end-of-life respiratory modalities on QODD and symptom relief was measured by multivariable linear regression using COT as a reference.

**Results:**

Among 177 patients analyzed for end-of-life respiratory modalities, 80 had a completed bereavement survey. The most common end-of-life respiratory modality was HFNC (n = 76, 42.9%), followed by COT (n = 62, 35.0%), NIV (n = 27, 15.3%), and IMV (n = 12, 6.8%). Regarding the place of death, 98.7% of patients treated with HFNC died outside the intensive care unit. Multivariable regression analyses revealed patients treated with HFNC had a higher GDI score for QODD [partial regression coefficient (B) = 0.46, 95% CI 0.07–0.86] and domain score related to symptom relief (B = 1.37, 95% CI 0.54–2.20) than those treated with COT.

**Conclusion:**

HFNC was commonly used in patients with end-stage ILD who died in the hospital and was associated with higher bereaved family ratings of QODD and symptom relief. HFNC might contribute to improved QODD and symptom relief in these patients who die in a hospital setting.

**Supplementary Information:**

The online version contains supplementary material available at 10.1186/s12931-022-02004-x.

## Introduction

Many types of interstitial lung diseases (ILDs), including idiopathic pulmonary fibrosis (IPF), are progressive, incurable diseases with poor prognosis [1, 2]. They are associated with distressing symptoms such as dyspnea, intractable cough, and fatigue that limit activity and impair quality of life (QOL). Patients with ILD suffer from severe dyspnea, which is more common and severe at the end of life [3–5]. Breathing comfort is a high end-of-life priority for terminally ill patients [6], and the management of dyspnea is a serious unmet need in palliative care for ILD patients. Morphine and benzodiazepines are often administered to these patients in clinical practice to manage symptoms including dyspnea [7, 8], but these medications were shown to be insufficiently effective for chronic breathlessness in a palliative care setting [9] and few reports have examined the effects of symptom relief in end-of-life settings.

The choice of end-of-life respiratory modalities [10–12], which may affect the quality of dying and death (QODD) of patients with ILD, is important to address dyspnea and the other symptoms at the end of life. Options include conventional oxygen therapy (COT), high-flow nasal cannula (HFNC), non-invasive ventilation (NIV), and invasive mechanical ventilation (IMV). Non-invasive respiratory support, such as HFNC and NIV, are increasingly used in end-of-life settings for terminally ill patients due to their comfort and the availability of oxygen. In particular, HFNC has attracted increasing attention in recent years in palliative settings [13, 14]. However, there have been few studies on end-of-life respiratory modalities applied to terminally ill ILD patients in clinical practice, and its impact on QODD and symptom relief are not fully understood.

The present multicenter descriptive cross-sectional study examined the proportion of end-of-life respiratory modalities applied to patients with end-stage ILD in real-world practice and assessed its impact on QODD and symptom relief from the perspective of the bereaved family.

## Methods

### Settings and procedures

A secondary analysis was conducted using data from a multicenter observational study including clinical and QODD data from patients dying with ILD and lung cancer [5]. This multicenter study was conducted at four major acute general hospitals in the western part of the Shizuoka Prefecture, Japan: Hamamatsu University Hospital, Seirei Mikatahara Hospital, Seirei Hamamatsu Hospital, and Iwata City Hospital. The inclusion criteria of this secondary analysis were patients with fibrotic ILD who died in the participating institutes from October 2015 to March 2019, age > 20 years, and patients with family members aged > 20 years. Exclusion criteria were family members who lacked the capacity to complete the questionnaire (due to dementia, cognitive failure, psychiatric illness, language difficulty, or vision loss) and family members who had severe emotional distress, as determined by their primarily responsible physician.

The medical records of the consecutive patients were reviewed, and information on baseline characteristics and medical interventions was collected. A cross-sectional bereavement survey was performed via mail to quantify the QODD from the bereaved family’s perspective between October and November 2019. The bereavement survey measurements were linked to the same patient’s information obtained from the medical records. This study was approved by the ethics board of Hamamatsu University School of Medicine and all participating institutions.

### End-of-life respiratory modalities

The primary exposure of this secondary analysis was end-of-life respiratory modalities received by patients who died of ILD. The respiratory interface applied to patients at the time of death was defined as end-of-life respiratory modalities. Clinical data on respiratory modalities were obtained from the medical records by one of the investigators and patients were divided according to respiratory modality into four groups: HFNC, COT, NIV, and IMV.

### QODD

The primary outcome was QODD, as rated using the Good Death Inventory (GDI), which is a validated and reliable tool for measuring QODD from the perspective of the bereaved family [15–17]. An overview of the questionnaire completed by the bereaved families is shown in Additional file 1: Table S1. It was developed based on qualitative interviews and a quantitative study of bereaved family members of deceased patients with cancer and consists of 18 domains, including 10 core and eight optional domains. Secondary outcome was “Physical and psychological comfort” domain (related to symptom relief) score. That is one of the GDI core domains that consists of three questions: “Patient was free from pain,” “Patient was free from physical distress,” and “Patient was free from emotional distress.” Bereaved family members were asked to rate the patient’s QODD in their final place of care using a seven-point Likert-type scale. Higher values indicated higher QODD.

### Statistical analysis

We first determined the proportion of end-of-life respiratory modalities among patients dying with ILD. The patients’ characteristics and medical interventions between each respiratory modality group were compared. Fisher’s exact test was used for categorical variables and one-way analysis of variance was used for quantitative variables. Summary statistics were calculated as numbers (with percentages), medians [with interquartile ranges (IQRs)], and means [with standard deviations (SDs)] as appropriate.

The impact of HFNC, NIV, and IMV (using COT as the reference group) on the outcome (i.e., GDI score for QODD) was calculated using univariable and multivariable linear regression analysis in patients with ILD whose bereaved families completed the questionnaire. COT was selected as the reference group as it has been widely prescribed for respiratory management of terminally ill patients for decades [18, 19], and we expected that this comparison would provide clinically interpretable insight. The primary outcome was calculated as the average score of 18 domains of the GDI and the secondary outcome was calculated as the score of “physical and psychological comfort” domain related to symptom relief. The choice of secondary outcome was based on the empirical finding that non-invasive respiratory support, such as HFNC and NIV, improved dyspnea in terminally ill patients and increased comfort [20, 21]. Therefore, we expected that these respiratory modalities would positively impact symptom relief. The following variables were selected as potential confounders based on findings from previous studies [22, 23] and the clinical perspective of the pulmonologist: patient’s age at death, patient’s sex, relationship between patient and family, cause of death (acute exacerbation or others), opioid use, and sustained sedation use. In addition, we evaluated the potential impact of medication (i.e., opioid use and sustained sedation) on the outcomes using the same multivariate regression model.

A two-sided test was used to determine significant differences, with the significance level set at *P* < 0.05. All statistical analyses were performed using EZR version 1.52 (Saitama Medical Centre, Jichi Medical University, Saitama, Japan) software, which is a graphical user interface for R version 4.02 (The R Foundation for Statistical Computing, Vienna, Austria) [24].

## Results

### Patients’ characteristics

A total of 177 consecutive patients died of ILD. The baseline characteristics of the patients are shown in Table 1. Patients with ILD included 137 men (77.4%) and 40 women (22.6%) with a mean age of 76 years. Overall, 92 patients (52.0%) used long-term oxygen therapy prior to final admission. ILD diagnoses included IPF (n = 78, 44.1%), idiopathic interstitial pneumonia excluding IPF (n = 58, 32.8%), interstitial pneumonia associated with connective tissue diseases (n = 36, 20.3%), chronic hypersensitivity pneumonitis (n = 3, 1.7%), and others (n = 2, 1.1%). Acute exacerbation was the most common cause of death (55.9%), followed by exacerbation of chronic respiratory failure (27.7%) and respiratory infection (8.5%). The median survival time from diagnosis was 31 months (IQR, 6–61 months).

**Table 1:** Patients’ characteristics and medical interventions at end of life

		Respiratory modality				p value
	All	HFNC	COT	NIV	IMV	
	n = 177	n = 76	n = 62	n = 27	n = 12	
Baseline characteristics						
Age, years	76.0 (8.3)	75.1 (8.8)	76.7 (7.9)	78.0 (8.6)	74.3 (6.6)	0.33
Sex, Male	137 (77.4)	63 (82.9)	46 (74.2)	19 (70.4)	9 (75.0)	0.48
LTOT, yes	92 (52.0)	40 (52.6)	39 (62.9)	11 (40.7)	2 (16.7)	0.02
Type of disease						0.15
IPF	78 (44.1)	43 (56.6)	24 (38.7)	10 (37.0)	1 (8.3)	
Non-IPF IIP	58 (32.8)	18 (23.7)	23 (37.1)	10 (37.0)	7 (58.3)	
CTD-IP	36 (20.3)	13 (17.1)	13 (21.0)	6 (22.2)	4 (33.3)	
CHP	3 (1.7)	2 (2.6)	1 (1.6)	0 (0.0)	0 (0.0)	
Others	2 (1.1)	0 (0.0)	1 (1.6)	1 (3.7)	0 (0.0)	
Cause of death						< 0.001
Acute exacerbation	99 (55.9)	54 (71.1)	18 (29.0)	18 (66.7)	9 (75.0)	
Exacerbation of chronic respiratory failure	49 (27.7)	9 (11.8)	33 (53.2)	7 (25.9)	0 (0.0)	
Respiratory infection	15 (8.5)	6 (7.9)	5 (8.1)	2 (7.4)	2 (16.7)	
Others	14 (7.9)	7 (9.2)	6 (9.7)	0 (0.0)	1 (8.3)	
End-of-life intervention						
Place of death						< 0.001
General wards	168 (94.9)	75 (98.7)	60 (96.8)	25 (92.6)	8 (66.7)	
ICU	8 (4.5)	1 (1.3)	1 (1.6)	2 (7.4)	4 (33.3)	
Hospice	1 (0.6)	0 (0.0)	1 (1.6)	0 (0.0)	0 (0.0)	
Opioids, yes	103 (58.2)	54 (71.1)	25 (40.3)	14 (51.9)	10 (83.3)	0.001
Sustained sedation, yes	40 (22.6)	15 (19.7)	7 (11.3)	8 (29.6)	10 (83.3)	< 0.001

Categorical variables were expressed as numbers (percentage). Quantitative variables were expressed as mean (SD). Fisher's exact test was used to analyze categorical variables, and one-way analysis of variance was used to analyze quantitative variables

HFNC, high-flow nasal cannula; COT, conventional oxygen therapy; NIV, non-invasive ventilation; IMV, invasive mechanical ventilation; LTOT, long-term oxygen therapy; IPF, idiopathic pulmonary fibrosis; Non-IPF IIP, idiopathic interstitial pneumonia excluding idiopathic pulmonary fibrosis; CTD-IP, connective tissue disease-related interstitial pneumonia; CHP, chronic hypersensitivity pneumonitis; ICU, intensive care unit; SD, standard deviation

### End-of-life respiratory modalities

**Fig. 1:**
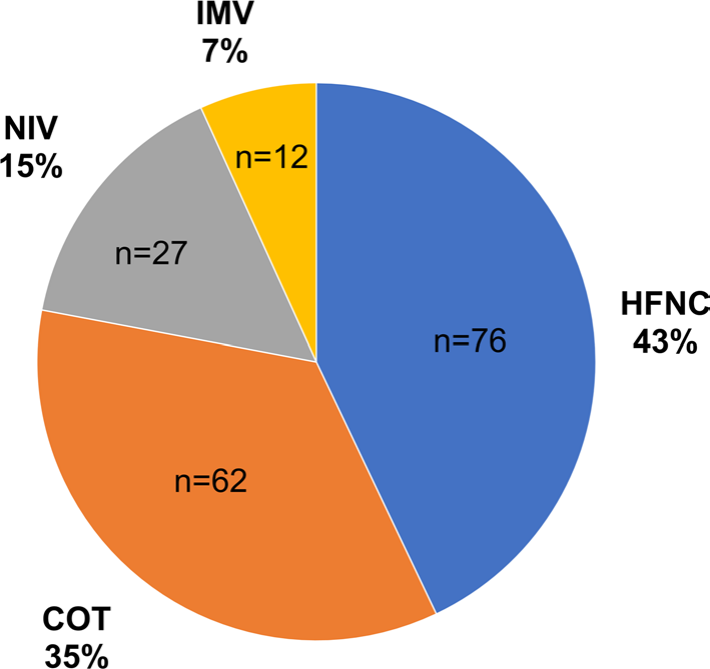
Proportion of end-of-life respiratory modalities among patients dying with ILD. COT, conventional oxygen therapy; HFNC, high-flow nasal cannula; ILD, interstitial lung disease; IMV, invasive mechanical ventilation; NIV, non-invasive ventilation

All patients included in the study were prescribed oxygen at the end of life. The most common end-of-life respiratory modality applied to patients with ILD was HFNC (n = 76, 42.9%), followed by COT (n = 62, 35.0%), NIV (n = 27, 15.3%), and IMV (n = 12, 6.8%) (Fig. 1). The ILD patients’ characteristics and medical interventions performed during the last hospitalization showed no significant differences between the groups in terms of age, sex, and type of ILD (Table 1). The COT group had a higher percentage of patients who died due to exacerbation of chronic respiratory failure compared with the other groups. Place of death was outside the intensive care unit (ICU) in 98.7% of patients treated with HFNC and 98.4% of those treated with COT. Patients treated with HFNC and IMV were more likely to be administered opioids than those treated with NIV and COT. Furthermore, 83% of patients treated with IMV received sustained sedation, while those treated with HFNC, COT, and NIV received less frequently.

### Impact of respiratory modalities on QODD and symptom relief

Among 177 patients with ILD, 80 whose bereaved families completed the questionnaire were analyzed to determine the impact of end-of-life respiratory modalities on QODD and symptom relief. The median time interval between patient death and the relatives' completion of the questionnaire was 24 months (IQR: 17–34 months). There were no significant differences in the characteristics and interventions between patients who responded to the questionnaire and those who did not (Additional file 1: Table S2). The patient characteristics and interventions for each group in QODD analysis population were similar to those in the whole population and there were no significant differences between the groups in terms of the characteristics of the bereaved family members and time interval between patient death and the relatives' completion of the questionnaire (Additional file 1: Table S3).

**Fig. 2:**
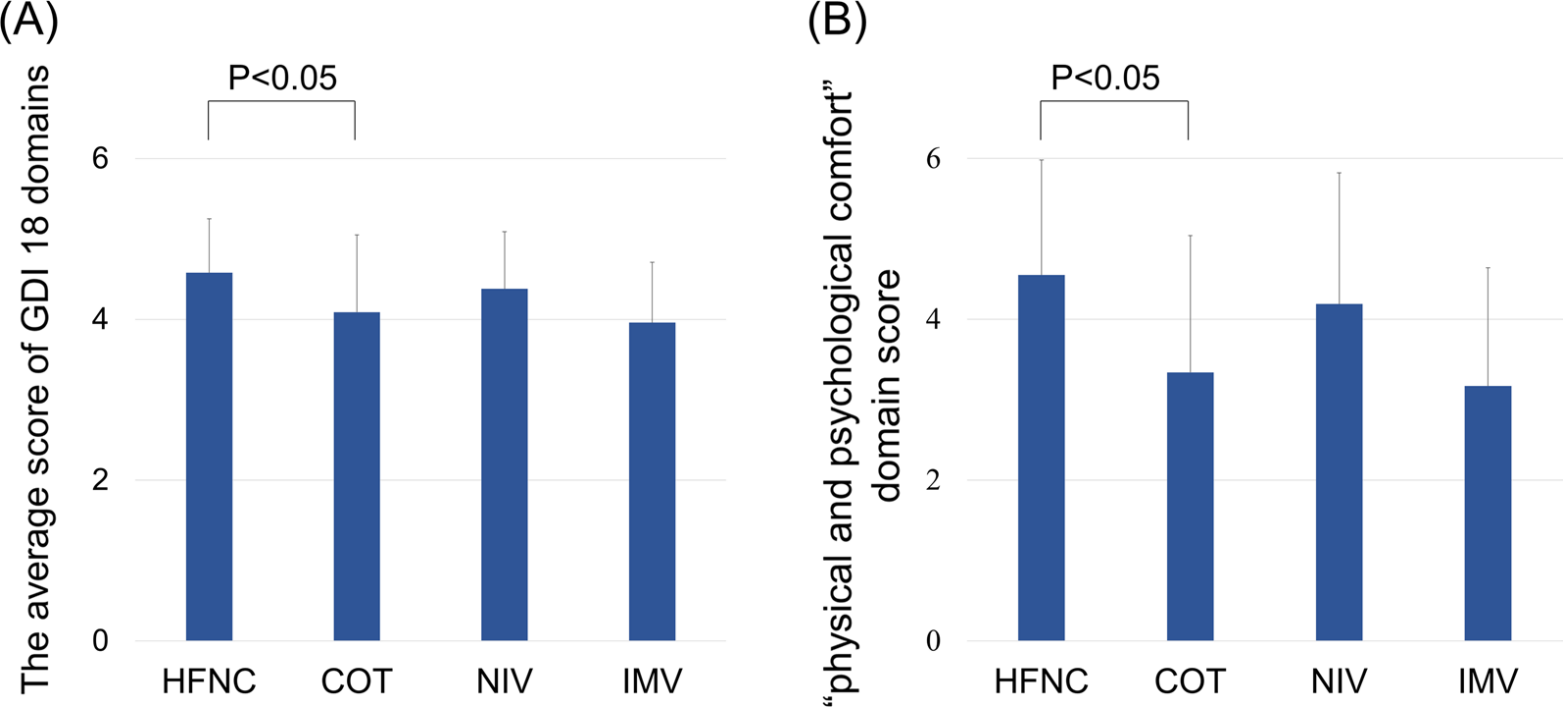
Average score of GDI 18 domains (**A**) and “physical and psychological comfort” domain (related to symptom relief) (**B**) for each end-of-life respiratory modality. Error bars indicate standard deviation. *P*-values were calculated by univariable linear regression analysis using COT as the reference group. COT, conventional oxygen therapy; GDI, Good death inventory; HFNC, high-flow nasal cannula; IMV, invasive mechanical ventilation; NIV, non-invasive ventilation

The average score of 18 domains of the GDI and the scores of each domain are presented in Additional file 1: Table S4, and the average score of the GDI and the “physical and psychological comfort” domain score are shown in Fig. 2. The average score of 18 domains of the GDI for QODD was the highest for the HFNC group (4.58 ± 0.67), followed by the NIV (4.38 ± 0.71), COT (4.09 ± 0.96), and IMV (3.96 ± 0.75) groups (Fig. 2A). Similarly, the score of the “physical and psychological comfort” domain was the highest for the HFNC group (4.55 ± 1.43), followed by the NIV (4.19 ± 1.63), COT (3.34 ± 1.70), and IMV (3.17 ± 1.47) groups (Fig. 2B). The mean GDI and “physical and psychological comfort” domain scores in patients treated with HFNC were significantly higher than those in patients treated with COT were.

Multivariable regression analyses were performed to investigate the impact of end-of-life respiratory modalities on QODD and symptom relief (Table 2). The QODD (average score of the 18 GDI domains) was significantly higher in patients treated with HFNC than in those treated with COT [partial regression coefficient (B) = 0.49, 95% CI 0.09–0.88, *P* = 0.02]. Additionally, the “physical and psychological comfort” domain score (related to symptom relief) was also significantly higher in patients treated with HFNC than those in patients treated with COT was (B = 1.43, 95% CI 0.59–2.26, *P* = 0.001). In contrast, this multivariate regression analysis showed that opioids and sustained sedation were not significantly associated with higher score of QODD and that of symptom relief.

**Table 2:** Results of multivariate regression analyses of scores of the Good death inventory for quality of dying and death for ILD patients treated with HFNC, NIV, or IMV, compared with those with COT (reference group)

	Average score of 18 domains of GDI			"Physical and psychological comfort" domain score		
	*B*	95% CI	p value	*B*	95% CI	p value
Respiratory modality (vs. COT)						
HFNC	0.49	0.09 to 0.88	0.02	1.43	0.59 to 2.26	0.001
NIV	0.34	−0.22 to 0.91	0.23	0.76	−0.43 to 1.95	0.21
IMV	0.01	−0.75 to 0.77	0.98	0.05	−1.56 to 1.66	0.95
Opioid use (vs. no use)	0.38	−0.05 to 0.80	0.08	-0.13	−1.03 to 0.77	0.77
Sustained sedation (vs. no use)	0.10	−0.36 to 0.55	0.67	0.28	−0.66 to 1.23	0.55

The scores of GDI for QODD for patients treated with HFNC, NIV, or IMV were tested using a multivariate linear regression model with those treated with COT as the reference group. This model included the patient’s age at death, patient’s sex, the relationship between the patient and the family, cause of death (i.e., acute exacerbation or others), respiratory modality, opioid use, and sustained sedation use as independent variable

*B*, partial regression coefficient; GDI, Good death inventory; HFNC, high-flow nasal cannula; COT, conventional oxygen therapy; NIV, non-invasive ventilation; IMV, invasive mechanical ventilation

## Discussion

The present multicenter study revealed that HFNC was the most commonly used end-of-life respiratory modality for patients with end-stage ILD in a hospital setting in Japan. Additionally, the use of HFNC at the end of life was associated with higher bereaved family ratings of QODD and higher ratings of symptom relief than the use of COT. To the best of our knowledge, this study is the first to explore the proportion of end-of-life respiratory modalities and the impact of end-of-life respiratory modalities on QODD and symptom relief in ILD patients dying in hospitals. These findings suggest that HFNC might contribute to improved QODD and symptom relief in these patients.

Fibrotic ILDs, represented by IPF, have a poor prognosis and are associated with dyspnea, cough, fatigue, anxiety, and depression. Among these symptoms, dyspnea is particularly distressing to patients with ILD [25], and high levels of dyspnea are associated with poor QOL [26]. Unfortunately, the frequency and severity of hypoxemia and dyspnea frequently increase near the end of life. Ahmadi et al. reported a higher prevalence of dyspnea in patients with oxygen-dependent ILD at the end of life, which was more refractory to treatment than that in patients with lung cancer [4]. Similarly, our multicenter observational study showed that the prevalence of very severe breathlessness was more than twofold greater in patients with ILD than patients with lung cancer (55.8% vs. 26.7%) [5]. These findings suggest that difficulties managing breathlessness and relief of dyspnea is an unmet need in the palliative care of patients with end-stage ILD. The pharmacological treatment of dyspnea is not well understood, and non-pharmacological approaches, including choice of respiratory modalities, have become a great concern [12]. Respiratory modalities at the end of life should be designed to improve patient comfort and maintain their communication abilities. An interface to provide a higher fraction of inspiratory oxygen is required to improve very severe hypoxemia in patients with end-stage ILD, and HFNC may be useful in the palliative care of patients with end-stage ILD; however, there is little evidence to support its use in this setting.

HFNC is a relatively new respiratory modality that can deliver high flow rates of 30–60 L/min of heated humidified gas at controlled oxygen concentrations via a large caliber nasal cannula [27]. HFNC has several characteristics that would greatly contribute to maintaining a better QOL for patients with end-stage ILD. First, it can relieve dyspnea caused by hypoxia since it allows the administration of a high flow and high concentration of oxygen. The FLORALI study reported a significantly better rate of improvement of dyspnea in the HFNC group than the NIV and COT groups [20], which is consistent with the present study results. Second, HFNC is a more favorable for terminally ill patients because it allows them to maintain daily activities such as eating and talking. Our previous study has revealed that patients treated with HFNC were able to maintain better oral intake and ability to converse until just before death compared with those treated with NIV among patients with ILD with a do-not-intubate order [28]. Additionally, the aforementioned study also found that HFNC was better tolerated and had fewer adverse events than NIV. While some issues, such as the cost-effectiveness of the use of HFNC in palliative care settings, remain to be examined, the results of the present study as well as previous studies highlight the use of HFNC as a reasonable and feasible palliative care treatment in patients with end-stage ILD.

The current study also showed that patients treated with IMV at the end of life had the lowest QODD score in the respiratory modality groups. Also, those treated with IMV died more frequently in the ICU. These findings raise the concern that a limit of life-sustaining care and location of death should be discussed earlier and more often in the disease. Patients with ILD have been reported to have an insufficient discussion and a poor understanding of disease behavior and prognosis [29]. Additionally, several studies reported that patients with ILD had a higher frequency of undecided life-sustaining care plans at the end of life than patients with lung cancer [5, 30]. Promoting discussion and shared decision making between patients, their families, and health care providers is important challenges that need to be addressed to help patients with ILD achieve a good death.

The present study has several limitations. First, this study was conducted on patients with ILD who died in the hospitals in Japan, not including patients who died at home or in nursing homes. It is well known that many patients with ILD prefer to die at home or in a hospice [31] and that the location of death varies by culture and geography [32, 33]. Therefore, these findings cannot be directly applicable to all patients with ILD. However, we believe the findings of this study have a degree of external validity since many patients with ILD, regardless of culture or region/countries, have been reported to die in a hospital setting [34]. Second, selection bias became a concern as the response rate was moderate (i.e., 45%). In addition, there was a recall and proxy bias in the bereavement survey. In this study, the period between the patient's death and the relatives' completion of the questionnaire was longer because ILDs are relatively rare diseases. Moreover, the nature of cross-sectional design cannot determine the causal relationship. Therefore, careful interpretation is required after integrating other results. Third, the number of patients treated with NIV or IMV was small. Studies that include a larger number of patients are required to evaluate the impact of these respiratory modalities. Fourth, we did not examine the sequence of respiratory management, the settings of each respiratory device, and adverse events. The association between these concerns and QODD needs to be studied in the future.

In conclusion, the present study showed that HFNC was the most commonly used end-of-life respiratory modality in patients dying with end-stage ILD in a hospital setting. Furthermore, HFNC application at the end of life was associated with higher bereaved family ratings of QODD than COT, as well as higher ratings of domain related to symptom relief. While prospective interventional studies are required to determine the optimal end-of-life respiratory modality, our findings suggest that HFNC might contribute to improved QODD and relief of distressing symptoms in patients with end-stage ILD who died in a hospital setting.

## Supplementary Information

**Additional file 1: Table S1.** Good Death Inventory (GDI). **Table S2.** Patients’ characteristics and medical interventions of patients with or without questionnaire response. **Table S3.** Patients’ characteristics, medical interventions, and bereaved family characteristics in QODD analysis population. **Table S4.** Good Death Inventory domain scores for quality of dying and death among patients with ILD.

## Data Availability

Participants of this study did not agree for their data to be shared publicly, so supporting data are not available.

## Abbreviations

QODD: Quality of dying and death
QOL: Quality of life
HFNC: High-flow nasal cannula
COT: Conventional oxygen therapy
NIV: Non-invasive ventilation
IMV: Invasive mechanical ventilation
ILD: Interstitial lung disease
IPF: Idiopathic pulmonary fibrosis
