# Supplementary material for: Impact of end-of-life respiratory modalities on quality of dying and death and symptom relief in patients with interstitial lung disease: a multicenter descriptive cross-sectional study
Source: Respir Res. 2022 Apr 4;23:79. doi: 10.1186/s12931-022-02004-x (PMC8981636; doi:10.1186/s12931-022-02004-x)
Supplement: Supplementary file 1 — Additional file 1: Table S1. Good Death Inventory (GDI). Table S2. Patients’ characteristics and medical interventions of patients with or without questionnaire response. Table S3. Patients’ characteristics, medical interventions, and bereaved family characteristics in QODD analysis population. Table S4. Good Death Inventory domain scores for quality of dying and death among patients with ILD. [file 12931_2022_2004_MOESM1_ESM.pdf]

Impact of end-of-life respiratory modalities on quality of dying and death and symptom relief in patients with interstitial lung disease: a multicenter descriptive cross-sectional study

Takafumi Koyauchi[1], Yuzo Suzuki[1], Kazuki Sato[2], Hironao Hozumi[1], Masato Karayama[1], Kazuki Furuhashi[1], Tomoyuki Fujisawa[1], Noriyuki Enomoto[1], Yutaro Nakamura[1], Naoki Inui[1], Koshi Yokomura[3], Shiro Imokawa[4], Hidenori Nakamura[5], Tatsuya Morita[6], Takafumi Suda[1]

[1]Second Division, Department of Internal Medicine, Hamamatsu University School of Medicine, Shizuoka, Japan

[2]Nursing for Advanced Practice, Division of Integrated Health Sciences, Nagoya University Graduate School of Medicine, Aichi, Japan

[3]Department of Respiratory Medicine, Respiratory Disease Centre, Seirei Mikatahara Hospital, Shizuoka, Japan.

[4]Department of Respiratory Medicine, Iwata City Hospital, Shizuoka, Japan

[5]Department of Respiratory Medicine, Seirei Hamamatsu Hospital, Shizuoka, Hamamatsu, Japan

[6]Department of Palliative and Supportive Care, Palliative Care Team and Seirei Hospice, Seirei Mikatahara Hospital, Shizuoka, Japan

Table S1. Good Death Inventory (GDI)

How do you think the patient felt during the end-of-life period? Please place the appropriate number next to each statement: 1: absolutely disagree, 2: disagree, 3: somewhat disagree, 4: unsure, 5: somewhat agree, 6: agree, 7: absolutely agree.	
**I. Physical and psychological comfort**	**II. Dying in a favorite place**
Patient was free from pain.	Patient was able to stay at his or her favorite place.
Patient was free from physical distress.	Patient was able to die at his or her favorite place.
Patient was free from emotional distress.	The place of death met the preference of the patient.
**III. Maintaining hope and pleasure**	**IV. Good relationship with medical staff**
Patient lived positively.	Patient trusted the physician.
Patient had some pleasure in daily life.	Patient had a professional nurse with whom he or she felt comfortable.
Patient lived in hope.	Patient had people who listened.
**V. Not being a burden to others**	**VI. Good relationship with family**
Patient was not being a burden to others (*).	Patient had family support.
Patient was not being a burden to family members (*).	Patient spent enough time with his or her family.
Patient had no financial worries (*).	Patient had family to whom he or she could express feelings.
**VII. Independence**	**VIII. Environmental comfort**
Patient was independent in moving or waking up.	Patient lived in quiet circumstances.
Patient was independent in daily activities.	Patient lived in calm circumstances.
Patient was not troubled with excretion.	Patient was not troubled by other people.
**IX. Being respected as an individual**	**X. Life completion**
Patient was not treated as an object or a child.	Patient had no regrets.
Patient was respected for his or her values.	Patient felt that his or her life was completed.
Patient was valued as a person.	Patient felt that his or her life was fulfilling.
**XI. Receiving enough treatment**	**XII. Natural death**
Patient received enough treatment.	Patient was not connected to medical instruments or tubes.
Patient believed that all available treatments were used.	Patient did not receive excessive treatment.
Patient fought against disease until the last moment.	Patient died a natural death.
**XIII. Preparation for death**	**XIV. Control over the future**
Patient met people whom he or she wanted to see.	Patient knew how long he or she was expected to live.
Patient felt thankful to people.	Patient knew what to expect about his or her condition in the future.
Patient was able to say what he or she wanted to dear people.	Patient participated in decisions about treatment strategy.
**XV. Unawareness of death**	**XVI. Pride and beauty**
Patient died without awareness that he or she was dying.	Patient felt burden of a change in his or her appearance (*).
Patient lived as usual without thinking about death.	Patient felt burden of receiving pity from others (*).
Patient was not informed of bad news.	Patient felt burden of exposing his or her physical and mental weakness to family (*).
**XVII. Feeling that one's life is worth living**	**XVIII. Religious and spiritual comfort**
Patient felt that he or she could contribute to others.	Patient was supported by religion.
Patient felt that his or her life is worth living.	Patient had faith.
Patient maintained his or her role in family or occupation.	Patient felt that he or she was protected by a higher power.
(*) Inverse items.	

Table S2. Patients' characteristics and medical interventions of patients with or without questionnaire response

	Respondents	Non-respondents	
	n =80	n = 97	p value
**Baseline characeristics**			
Age, years	75.4 (8.7)	76.6 (8.0)	0.36
Sex, Male	66 (82.5)	71 (73.2)	0.15
LTOT, yes	45 (56.2)	47 (48.5)	0.37
Type of disease			0.67
IPF	36 (45.0)	42 (43.3)	
Non-IPF IIP	24 (30.0)	34 (35.1)	
CTD-IP	18 (22.5)	18 (18.6)	
CHP	2 (2.5)	1 (1.0)	
Others	0 (0.0)	2 (2.1)	
Cause of death			0.63
Acute exacerbation	47 (58.8)	52 (53.6)	
Exacerbation of chronic respiratory failure	22 (27.5)	27 (27.8)	
Respiratory infection	7 (8.8)	8 (8.2)	
Others	4 (5.0)	10 (10.3)	
**End-of-life Intervention**			
End-of-life respiratory modality			0.62
HFNC	36 (45.0)	40 (41.2)	
COT	29 (36.2)	33 (34.0)	
NIV	9 (11.2)	18 (18.6)	
IMV	6 (7.5)	6 (6.2)	
Place of death			0.59
General wards	76 (95.0)	92 (94.8)	
ICU	3 (3.8)	5 (5.2)	
Hospice	1 (1.2)	0 (0.0)	
Opioids, yes	48 (60.0)	55 (56.7)	0.76
Sustained sedation, yes	20 (25.0)	20 (20.6)	0.59

Categorical variables were expressed as numbers (percentage). Quantitative variables were expressed as mean (SD). Fisher's exact test was used to analyze categorical variables, and the Student's *t*-test was used to analyze quantitative variables.

LTOT, long-term oxygen therapy; IPF, idiopathic pulmonary fibrosis; Non-IPF IIP, idiopathic interstitial pneumonia excluding idiopathic pulmonary fibrosis; CTD-IP, connective tissue

disease-related interstitial pneumonia; CHP, chronic hypersensitivity pneumonitis; HFNC, high-flow nasal cannula; COT, conventional oxygen therapy; NIV, non-invasive ventilation; IMV, invasive mechanical ventilation; ICU, intensive care unit; SD, standard deviation.

Table S3. Patients' characteristics, medical interventions, and bereaved family characteristics in QODD analysis population

	Respiratory modality				
	HFNC	COT	NIV	IMV	
	n =36	n = 29	n = 9	n = 6	p value
**Baseline characeristics**					
Age, years	74.5 (9.7)	75.8 (7.7)	78.1 (9.2)	75.0 (7.9)	0.74
Sex, Male	33 (91.7)	22 (75.9)	7 (77.8)	4 (66.7)	0.25
LTOT, yes	23 (63.9)	17 (58.6)	3 (33.3)	2 (33.3)	0.25
Type of disease					0.08
IPF	23 (63.9)	9 (31.0)	3 (33.3)	1 (16.7)	
Non-IPF IIP	7 (19.4)	12 (41.4)	3 (33.3)	2 (33.3)	
CTD-IP	4 (11.1)	8 (27.6)	3 (33.3)	3 (50.0)	
CHP	2 (5.6)	0 (0.0)	0 (0.0)	0 (0.0)	
Cause of death					0.049
Acute exacerbation	25 (69.4)	12 (41.4)	5 (55.6)	5 (83.3)	
Exacerbation of chronic respiratory failure	5 (13.9)	13 (44.8)	4 (44.4)	0 (0.0)	
Respiratory infection	5 (13.9)	1 (3.4)	0 (0.0)	1 (16.7)	
Others	1 (2.8)	3 (10.3)	0 (0.0)	0 (0.0)	
**End-of-life Intervention**					
Place of death					<0.001
General wards	36 (100.0)	28 (96.6)	9 (100.0)	3 (50.0)	
ICU	0 (0.0)	0 (0.0)	0 (0.0)	3 (50.0)	
Hospice	0 (0.0)	1 (3.4)	0 (0.0)	0 (0.0)	
Opioids, yes	29 (80.6)	12 (41.4)	2 (22.2)	5 (83.3)	0.001
Sustained sedation, yes	9 (25.0)	4 (13.8)	2 (22.2)	5 (83.3)	0.005
**Bereaved family members**					
Age, years					0.11
> 49	6 (16.7)	2 (6.9)	1 (11.1)	2 (33.3)	
50-59	8 (22.2)	4 (13.8)	0 (0.0)	2 (33.3)	
60-69	8 (22.2)	9 (31.0)	6 (66.7)	2 (33.3)	
>= 70	14 (38.9)	14 (48.3)	2 (22.2)	0 (0.0)	
Sex, male	10 (27.8)	8 (27.6)	1 (11.1)	4 (66.7)	0.13
Relationship to patient					0.26
Husband/wife	22 (61.1)	20 (69.0)	5 (55.6)	1 (16.7)	
Child of patient	12 (33.3)	8 (27.6)	4 (44.4)	5 (83.3)	
Others	2 (5.6)	1 (3.4)	0 (0.0)	0 (0.0)	
Frequency of visits during the last hospitalization					0.99
everyday	24 (66.7)	21 (72.4)	7 (77.8)	4 (66.7)	
4-6 days per week	4 (11.1)	2 (6.9)	1 (11.1)	1 (16.7)	
1-3 days per week	6 (16.7)	5 (17.2)	1 (11.1)	1 (16.7)	
no visit	2 (5.6)	1 (3.4)	0 (0.0)	0 (0.0)	
Time between the patient death and the questionnaire completion, months	23 (17-36)	27 (16-35)	18 (17-22)	29.5 (29-30)	0.18

Categorical variables were expressed as numbers (percentage). Quantitative variables were expressed as mean (SD) or median (IQR). Fisher's exact test was used to analyze categorical variables, and one-way analysis of variance or Kruskal-Wallis test was used to analyze quantitative variables as appropriate.

QODD, quality of dying and death; HFNC, high-flow nasal cannula; COT, conventional oxygen therapy; NIV, non-invasive ventilation; IMV, invasive mechanical ventilation; LTOT, long-term oxygen therapy; IPF, idiopathic pulmonary fibrosis; Non-IPF IIP, idiopathic interstitial pneumonia excluding idiopathic pulmonary fibrosis; CTD-IP, connective tissue disease-related interstitial pneumonia; CHP, chronic hypersensitivity pneumonitis; ICU, intensive care unit; SD, standard deviation; IQR, interquartile range.

Table S4. Good Death Inventory domain scores for quality of dying and death among patients with ILD

	HFNC<br>n = 36	COT<br>n = 29	NIV<br>n = 9	IMV<br>n = 6
Quality of death and dying (average of 18 domains of GDI)	4.58 (0.67)	4.09 (0.96)	4.38 (0.71)	3.96 (0.75)
Score of each domain				
Physical and psychological comfort	4.55 (1.43)	3.34 (1.70)	4.19 (1.63)	3.17 (1.47)
Dying in a favorite place	4.38 (1.54)	4.46 (1.79)	4.19 (1.89)	3.83 (1.24)
Maintaining hope and pleasure	4.10 (1.34)	3.26 (1.60)	3.59 (1.26)	4.06 (1.58)
Good relationship with medical staff	5.25 (1.11)	4.97 (1.28)	5.41 (0.91)	4.50 (1.39)
Not being a burden to others	4.00 (1.41)	3.85 (1.74)	4.30 (1.21)	3.78 (1.67)
Good relationship with family	4.96 (1.22)	4.69 (1.34)	4.93 (1.23)	4.61 (0.80)
Independence	4.10 (1.74)	3.47 (1.72)	3.37 (2.22)	3.61 (1.27)
Environmental comfort	5.02 (1.27)	4.43 (1.58)	4.11 (1.74)	4.50 (1.49)
Being respected as an individual	5.84 (0.78)	5.07 (1.70)	5.71 (0.93)	5.56 (0.91)
Life completion	4.19 (1.69)	3.79 (1.76)	4.26 (1.56)	3.72 (1.69)
Receiving enough treatment	5.31 (1.20)	4.43 (1.67)	5.26 (0.80)	4.39 (1.78)
Natural death	5.20 (1.15)	4.70 (1.39)	4.56 (1.49)	4.06 (0.93)
Preparation for death	5.02 (1.52)	4.49 (1.75)	4.70 (1.41)	4.56 (0.81)
Control over the future	4.28 (1.72)	4.26 (1.68)	4.19 (1.32)	2.94 (1.22)
Unawareness of death	3.96 (1.34)	3.57 (1.35)	4.33 (1.17)	3.28 (1.45)
Pride and beauty	3.75 (1.20)	3.61 (1.16)	4.15 (1.20)	3.33 (0.87)
Feeling that one's life is worth living	5.47 (1.32)	5.10 (1.46)	5.19 (1.37)	4.72 (1.24)
Religious and spiritual comfort	3.02 (1.71)	2.08 (2.04)	2.59 (2.41)	2.67 (1.05)

Quantitative variables were expressed as mean (SD). Good death inventory domain scores range from 1 to 7; higher scores indicate a higher perceived quality of dying and death.

GDI, Good Death Inventory; HFNC, high-flow nasal cannula; COT, conventional oxygen therapy; NIV, non-invasive ventilation; IMV, invasive mechanical ventilation; SD, standard deviation.
